# Copolymer Brush Particle Hybrid Materials with “Recall-and-Repair” Capability

**DOI:** 10.1021/acs.chemmater.3c01234

**Published:** 2023-08-17

**Authors:** Yuqi Zhao, Hanshu Wu, Rongguan Yin, Chenxi Yu, Krzysztof Matyjaszewski, Michael R. Bockstaller

**Affiliations:** † Department of Materials Science & Engineering, Carnegie Mellon University, 5000 Forbes Avenue, Pittsburgh, Pennsylvania 15213, United States; ‡ Department of Chemistry, Carnegie Mellon University, 4400 Fifth Avenue, Pittsburgh, Pennsylvania 15213, United States

## Abstract

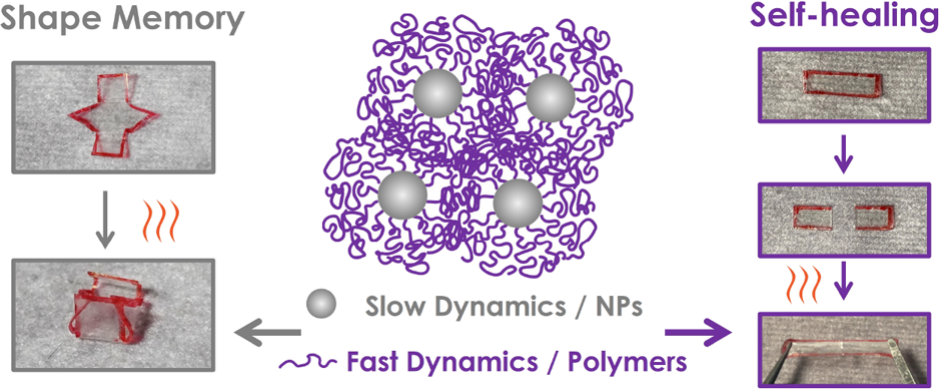

The effect of sequence structure on the self-healing and shape-memory properties of copolymer-tethered brush particle films was investigated and compared to linear copolymer analogs. Poly(*n*-butyl acrylate-*co*-methyl methacrylate), P(BA-*co*-MMA), and linear and brush analogs with controlled gradient and statistical sequence were synthesized by atom transfer radical polymerization (ATRP). The effect of sequence on self-healing in BA/MMA copolymer brush particle hybrids followed similar trends as for linear analogs. Most rapid restoration of mechanical properties was found for statistical copolymer sequence; an increase of the high *T*_g_ (MMA) component provided a path to raise the material’s modulus while retaining self-heal ability. Creep testing revealed profound differences between linear and brush systems. While linear copolymers featured substantial viscous deformation when exposed to constant stress in the linear regime, brush analogs displayed minimal permanent deformation and featured shape restoration. The reduction of flow was interpreted to be a consequence of slow cooperative relaxation due to the complex microstructure of brush particle hybrids in which long-range motions are constrained through entanglements and slow-diffusing particle cores. The rubbery-like response imparts BA/MMA copolymer brush material systems concurrent “shape-memory” and “self-heal” capability. This ability to “recall-and-repair” could find application in the design of functional hybrid materials, for example, for soft robotics.

## Introduction

Soft materials such as polymers are susceptible to structural damage either by direct load application or by external factors that cause embrittlement, cracking, and eventual functional degradation.^1−3^ This limits the lifetime of polymer parts and adds to polymer-based technologies’ economic and environmental cost.^4−7^ To alleviate this challenge, much research has focused on the development of polymer materials that can recover functional performance after incurring (minor) structural damage. Such “self-healing” materials could provide a path toward more sustainable polymer technologies with increased reliability and prolonged service life.^8,9^ Common approaches to realizing “self-heal ability” rely on the implementation of bio-inspired features such as embedded monomer reservoirs or polymer chemistries involving reversible primary and/or secondary bond network structures.^10−18^ However, while such “bio-inspired” approaches have shown promise, the cost of fabricating complex structured systems as well as the sensitivity of reversible bond chemistries to external parameters such as humidity remains a challenge.^19−21^ A novel concept for realizing self-heal ability in more commonly used engineering polymer platforms relies on the synthesis of copolymers with pendant groups capable of forming toothed wheel-like interlocked microstructures that promote van-der-Waals-driven structure reconstitution.^22,23^ This was first shown by Urban and coworkers who demonstrated that poly(*n*-butyl acrylate-*co*-methyl methacrylate) (P(BA-*co*-MMA)) copolymers with sufficient content of BA-MMA dyads featured self-heal ability.^18^ The deliberate variation of sequence structure in the case of P(BA-*co*-MMA) copolymers using controlled and free radical methods further confirmed that alternating or statistical sequence copolymers featured a significant acceleration of property recovery as compared to gradient analogs.^24,25^ Despite these advances, a common challenge to both, bio-inspired and van-der-Waals-driven self-healing, has been that the healing process is contingent on sufficient chain mobility to allow for the re-establishment of a bond network structure. This implies that structure recovery is tied to sufficiently high chain dynamics and thus low cohesive energy density and elastic modulus (<1 GPa) to avoid impractically high healing temperatures.^26−28^

An intriguing strategy to extend self-healing to higher-modulus polymer materials is the integration of shape-memory functionality to afford an additional energetic driving force for the healing process.^29−31^ Traditional shape-memory polymers employ a hierarchical network structure. A primary (permanent) network—based, for example, on covalent crosslinks—is used to define a permanent shape. A secondary (temporary) network—based, for example, on interlocked crystalline regions, reversible bonds, etc.—enables the fixation of a “new” shape upon the deformation of a polymer.^32,33^ Upon application of a stimulus (such as heating), the temporary network structure resolves, and the release of stored strain energy results in the recovery of the original (permanent) shape. The possibility to leverage stored strain energy in addition to surface energy to drive structure recovery holds the prospect of accelerating healing processes or enabling healing in systems with higher cohesive energy density (and thus slower dynamics or higher modulus).^32−37^ The possibility to harness viscoelastic effects—rather than permanent crosslink structures—to drive shape restoration has further expanded the applicability of the concept of “shape-memory” polymers.^38−42^ Thus, the requirement for a polymer to display shape-memory functionality is the presence of dynamical modes that are sufficiently slow to be considered static (on the timescale relevant for the application) and with the capacity of storing the required strain energy to enable shape restoration upon a suitable stimulus.^32,39,43^

Due to their microstructure, brush particles present a unique platform to facilitate the fabrication of shape-memory materials.^44−46^ Calorimetric as well as Brillouin light scattering studies of the dynamical properties of brush particle films revealed that the global dynamics (which is relevant to macroscopic shape fixation and restoration) is dominated by long-ranged cooperative relaxations that are absent in linear polymer systems.^47−50^ This was attributed to the more extensive coupling between entanglement points and the low mobility of particle cores in brush particle melts.^42^ First reports of brush particle-based shape-memory hybrid materials were due to Vaia and coworkers who demonstrated shape programming and reversal efficacies of 95% for polystyrene (PS)-grafted titania and silica brush particle films in the sparse grafting limit.^51−58^ Koerner et al. subsequently demonstrated that the slow relaxation dynamics in brush materials enable the use of higher shape-setting temperatures (relative to the glass transition temperature, *T*_g_) without loss of recovery.^51^ Common to these previous studies is that the amorphous polymer featured a high glass transition temperature (*T*_g,PS_ ≈ 105 °C), and thus shape setting could be accomplished by vitrification at ambient conditions. Here, we demonstrate that shape-memory properties can also be accomplished in densely grafted copolymer brush particle assemblies based on P(BA-*co*-MMA)-tethered silica particles that feature a glass transition below room temperature. Interestingly, the brush particle films retain the ability to “self-heal”, which is characteristic of the linear copolymer analogs. Like linear analogs, we find that raising the fraction of MMA in statistical sequence copolymers enables the (approximately) 10-fold increase of elastic modulus while retaining self-heal ability. This suggests a new path toward hybrid materials with “recall-and-repair” capability in which “self-healing” and “shape-memory” functionality can be synergistically combined, for example, to enable healing in high-modulus polymer material systems.

## Results and Discussion

**Figure 1:**
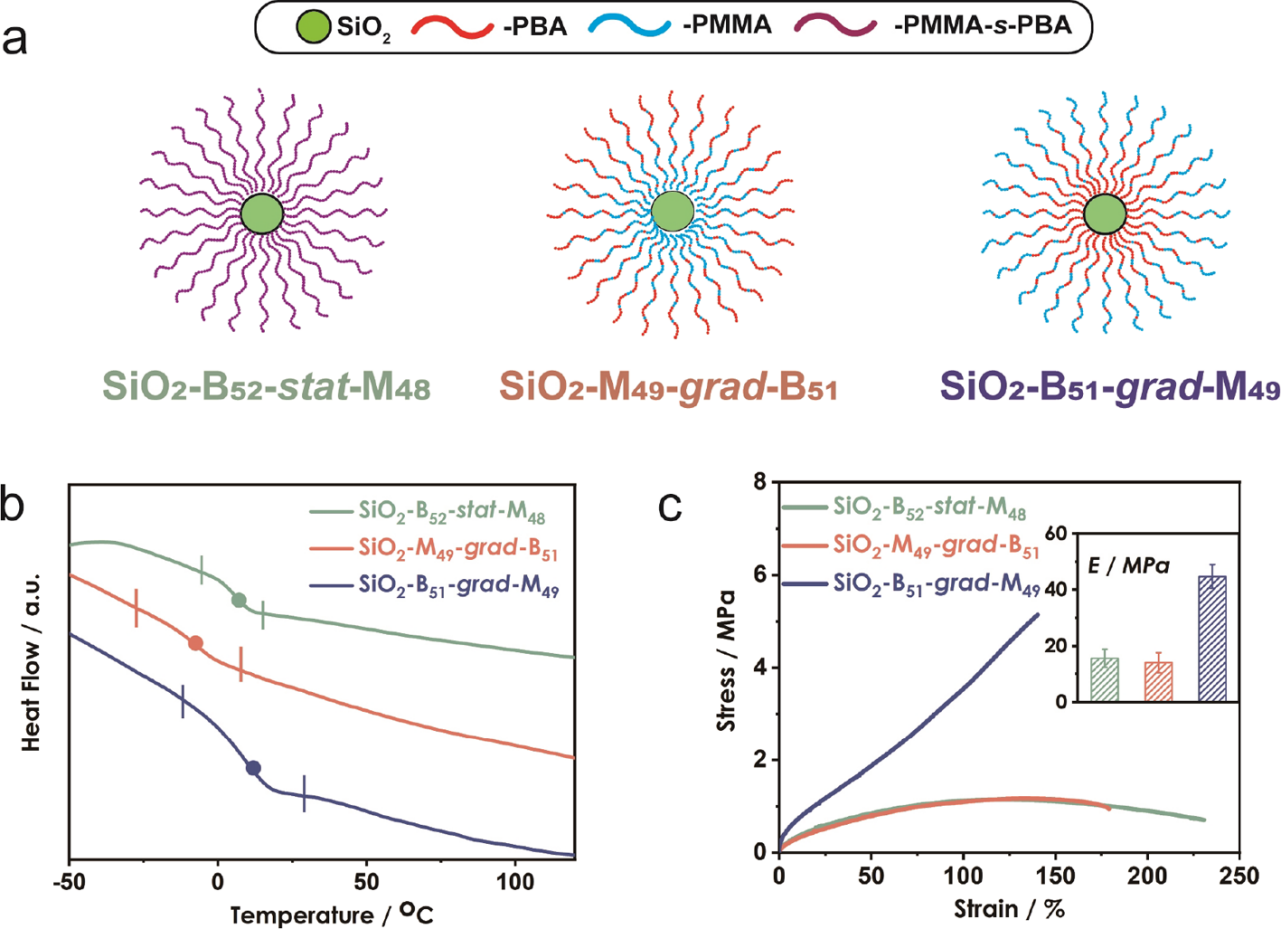
(a) Illustration of chain architecture in particle brush systems. (b) DSC heat flow curves; the position of *T*_g_’s are highlighted with solid points in the figures. The onset and offset points of the glass transitions are highlighted in the figures. Measurements were performed at the third heating cycle at a heating rate of 20 °C/min. (c) Strain–stress curves; inset: Young’s modulus calculated from the incipient slope of strain stress curves. Measurements were performed at a strain rate of 0.05 mm/mm/s.

Surface-initiated atom transfer radical polymerization (SI-ATRP) in the low conversion limit or semi-batch form was used to synthesize three types of poly(BA-*co*-MMA)-grafted silica brush particle systems with symmetric compositions (∼49 mol % MMA), similar molecular weight, and controlled sequence structure: statistical, SiO_2_-B_Y_-*stat*-M_Z_; gradient with an MMA-rich region outside (MMA-out), SiO_2_-B_Y_-*grad*-M_Z_; and gradient with a BA-rich region outside (BA-out), SiO_2_-M_Z_-*grad*-B_Y_; as well as their respective linear analogs.^59−62^ The particle brush systems will be abbreviated as SiO_2_-B_Y_-X-M_Z_, where SiO_2_ is the particle composition; X = *stat*/*grad* indicates statistical (*stat*) or gradient (*grad*) chain sequence; and Y and Z are the molar compositions of B (BA) and M (MMA), respectively. For linear analogs, the prefix “SiO_2_” is dropped. Note that for gradient sequence, two orientations are distinguished, i.e., MMA-out (B_Y_M_Z_) or BA-out (M_Z_B_Y_), depending on which component was polymerized first. The synthesis of MMA-out involved slow feeding of MMA monomers into BA solution, while BA-out was accomplished by slow feeding of BA monomers into solution of MMA (the detailed procedure is described in the Supporting Information). Structures of the different material systems are illustrated in Figure 1a. Synthesis and relevant molecular and compositional details are presented in Table 1.

**Table 1:** Characteristics of SiO_2_-*g*-P(BA-*stat*/*grad*-MMA) Copolymer Brush Particle Systems

entry^a^	***x***,_BA_ (mol %)^b^	***x***,_MMA_ (mol %)^b^	*M*_n_^c^	*M*_w_/*M*_n_^c^	*f*_SiO2_^d^ (%)	σ^e^ (nm^–2^)	*T*_g_^f^ (°C)	*T*_g_ range^f^ (°C)
SiO_2_-B_52_-*stat*-M_48_	51.7	48.3	27,600	1.21	18.3	0.56	6	[−7, 15]
SiO_2_-B_51_-*grad*-M_49_	50.7	49.3	30,720	1.19	18.6	0.50	9	[−18, 27]
SiO_2_-M_49_-*grad*-B_51_	51.4	48.6	39,610	1.16	15.1	0.49	–7	[−25, 10]
B_45_-*stat*-M_55_	45.3	54.7	32,470	1.14	NA	NA	16	[−6, 26]
SiO_2_-B_45_-*stat*-M_55_	45.3	54.7	31,340	1.20	16.5	0.56	19	[0, 27]

a Reaction conditions are listed in Experimental Section.

b Determined by ^1^H-NMR.

c Determined by SEC.

d Determined by thermogravimetric analysis (TGA).

e Calculated by eq S1.

f The glass transition temperature determined by DSC. Nanoparticle diameter *d* ∼ 15.5 ± 3.7 nm.

The effect of copolymer sequence structure on the thermomechanical behavior was characterized by differential scanning calorimetry (DSC) and tensile testing. Heat flow and derivative heat flow curves, shown in Figure 1 and Figure S1, revealed the impact of sequence on glass transition temperature for symmetric compositions. Like linear analogs, statistical sequence systems exhibited the narrowest *T*_g_ range (22 °C), compared to MMA-out (45 °C) and BA-out (35 °C) gradient systems, suggesting a more uniform microstructure (Figure 1b).^23,24^ Note that, contrary to linear analogs, the thermal analysis revealed a sensitive dependence of the glass transition on the brush orientation. The highest *T*_g_ was observed for MMA-out gradient SiO_2_-B_51_-*grad*-M_49_ (9 °C) as compared to the statistical SiO_2_-B_52_-*stat*-M_48_ (6 °C), and BA-out gradient SiO_2_-M_49_-*grad*-B_51_ (−7 °C). Interpretation of this effect is difficult as it is likely the cumulative result of a number of parameters such as the impact of steric confinement within the inner (concentrated) brush regions on chain relaxation and brush interdigitation or the detailed compositional gradient associated with each material system.^63^ However, our results suggest that a higher glass transition is observed for systems in which the high *T*_g_ component (i.e., MMA) is concentrated within the outer brush region and thus able to assume a continuous structure similar to the unperturbed linear state. Consistent with this observation was that SiO_2_-B_51_-*grad*-M_49_ also featured a higher Young’s modulus (44.7 MPa) in small strain deformation (see Figure 1c) as compared to statistical and BA-out gradient particle brush analogs (15.6 and 14.0 MPa). It is also notable that MMA-out systems were the only ones to feature strain-hardening behavior during large strain deformation.

The self-healing capabilities of the various brush particle films were tested by cut-and-adhere experiments following previously published procedures.^23^ To quantitively evaluate the healing efficiency, a severed bulk film was rejoined at 50 °C and annealed at 100 °C for a defined time before cooling to room temperature (testing conditions were chosen for practicality). Self-heal efficacy was determined by evaluating the fractional recovery of the elastic modulus (*P*_E_) and fracture toughness (*P*_U_) using tensile testing (see Figure S2). As a measure to evaluate the efficiency of recovery, the half-time τ_1/2_ is defined as time to recover 50% of the initial performance of specific properties.^23^

**Figure 2:**
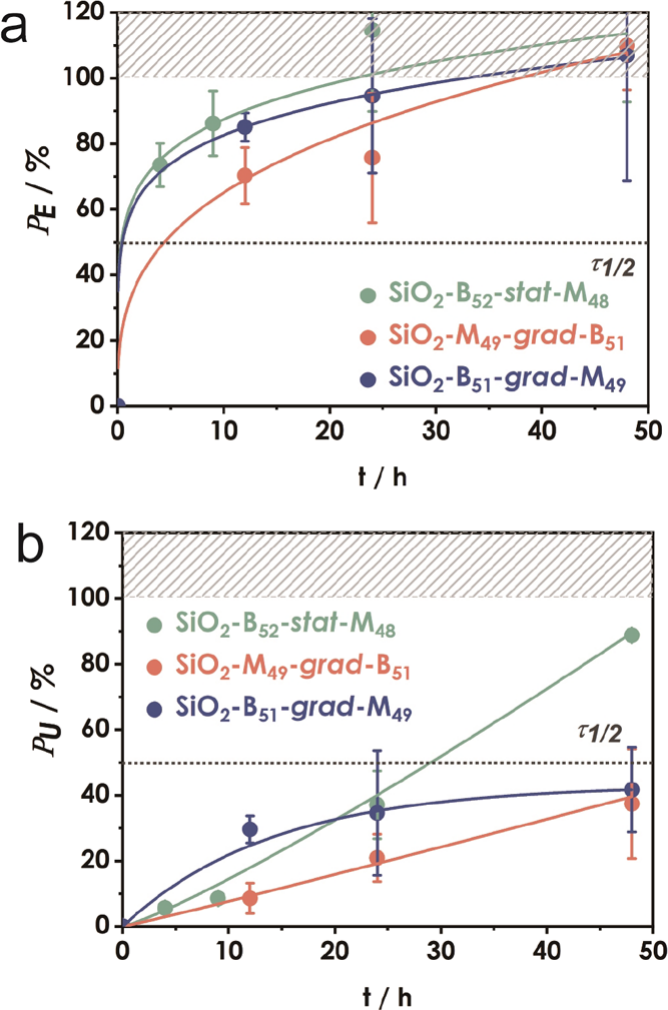
Property recovery of SiO_2_-B_52_-*stat*-M_48_, SiO_2_-M_49_-*grad*-B_51_, and SiO_2_-B_51_-*grad*-M_49_ after re-joining of films at 50 °C and subsequent annealing at 100 °C. Panels show fractional recovery of (a) Young’s modulus (*P*_E_) and (b) toughness (*P*_U_). Values in (a) and (b) are normalized with respect to pristine film properties. Lines are introduced to guide the eye.

Figure 2 reveals two pertinent observations: First, the recovery of both modulus and toughness was more efficient in the case of the statistical sequence structure of the copolymer. This bears similarity to the self-healing behavior of linear copolymer analogs for which more rapid recovery was found in the case of statistical (and alternating) sequence and attributed to a more uniform microstructure.^23,24^ Irrespective of sequence, the recovery of modulus was more rapid as compared to toughness for which only partial recovery was observed. This was rationalized as a consequence of the more long-range diffusion processes required to re-establish the entanglement network structures that determine the fracture toughness of polymers.^64−68^ Unexpectedly, SiO_2_-M_49_-*grad*-B_51_, with a soft BA-rich region in the periphery of the brush (i.e., BA-out), displayed the slowest recovery of both modulus and toughness. This indicates that the impact of brush architecture on self-heal efficacy is complex and dependent on a variety of parameters. We hypothesize that in the case of SiO_2_-M_49_-*grad*-B_51_, the inner (high *T*_g_) MMA-rich region increased the “effective particle size”, thus decreasing the mobility of the brush particles.

**Figure 3:**
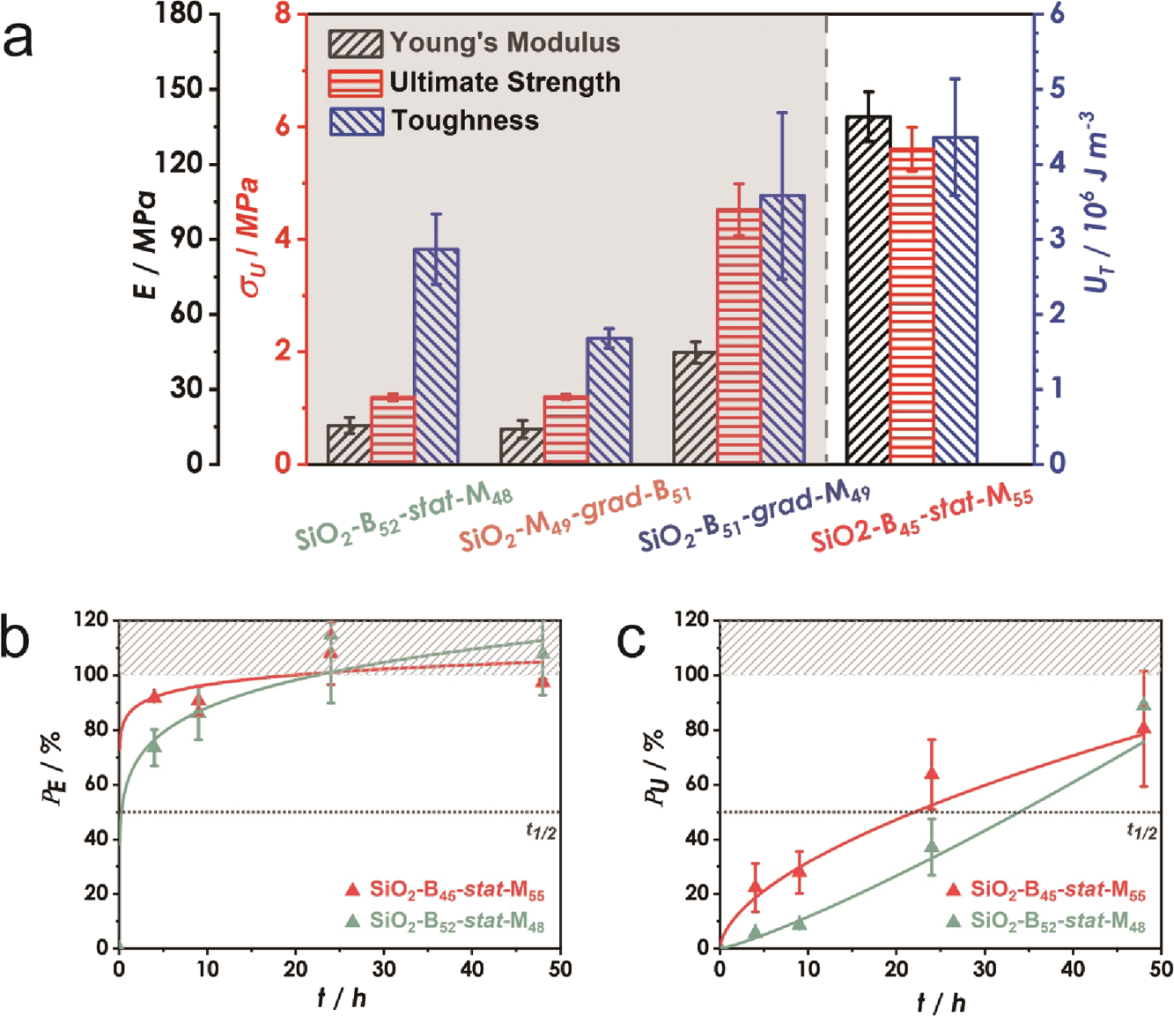
(a) Comparison of mechanical properties (Young’s modulus, ultimate strength, toughness) of SiO_2_-B_Y_-X-M_Z_ films determined by uniaxial tension testing. Self-healing is observed in *stat* particle brush SiO_2_-B_45_-*stat*-M_55_ despite the increased fraction of the glassy (MMA) component. Property recovery of SiO_2_-B_45_-*stat*-M_55_ and SiO_2_-B_52_-*stat*-M_48_ after re-joining of films at 100 °C for (b) Young’s modulus (*P*_E_) and (c) toughness (*P*_U_). Values in (b) and (c) are normalized with respect to pristine film properties. Lines are introduced to guide the eye.

Recently, we demonstrated that increasing the high *T*_g_ component in statistical P(BA-*stat*-MMA) copolymers enables efficient self-healing in polymers with increased elastic modulus.^23^ To test this concept for brush particle systems, a nonsymmetric composition of brush particles with statistical sequence (SiO_2_-B_45_-*stat*-M_55_) was tested. As shown in Figure 3a, SiO_2_-B_45_-*stat*-M_55_ featured a significantly higher Young’s modulus (139.0 MPa), ultimate strength (5.6 MPa), and toughness (4.36 MJ/m^3^), as compared to SiO_2_-B_52_-*stat*-M_48_ (15.6 MPa; 1.19 MPa; 2.87 MJ/m^3^) and SiO_2_-B_51_-*grad*-M_49_ (44.7 MPa; 4.53 MPa; 3.58 MJ/m^3^). Interestingly, increasing the fraction of MMA resulted in a faster recovery as compared to SiO_2_-B_52_-*stat*-M_48_ (although the difference is somewhat blurred by the uncertainty of the measurement; Figure 3b,c). We hypothesize that this intriguing and counterintuitive result could be attributed to the number of accessible BA/MMA dyads. However, experimental validation of this hypothesis is difficult and thus further research will have to be performed to elucidate this aspect.

**Figure 4:**
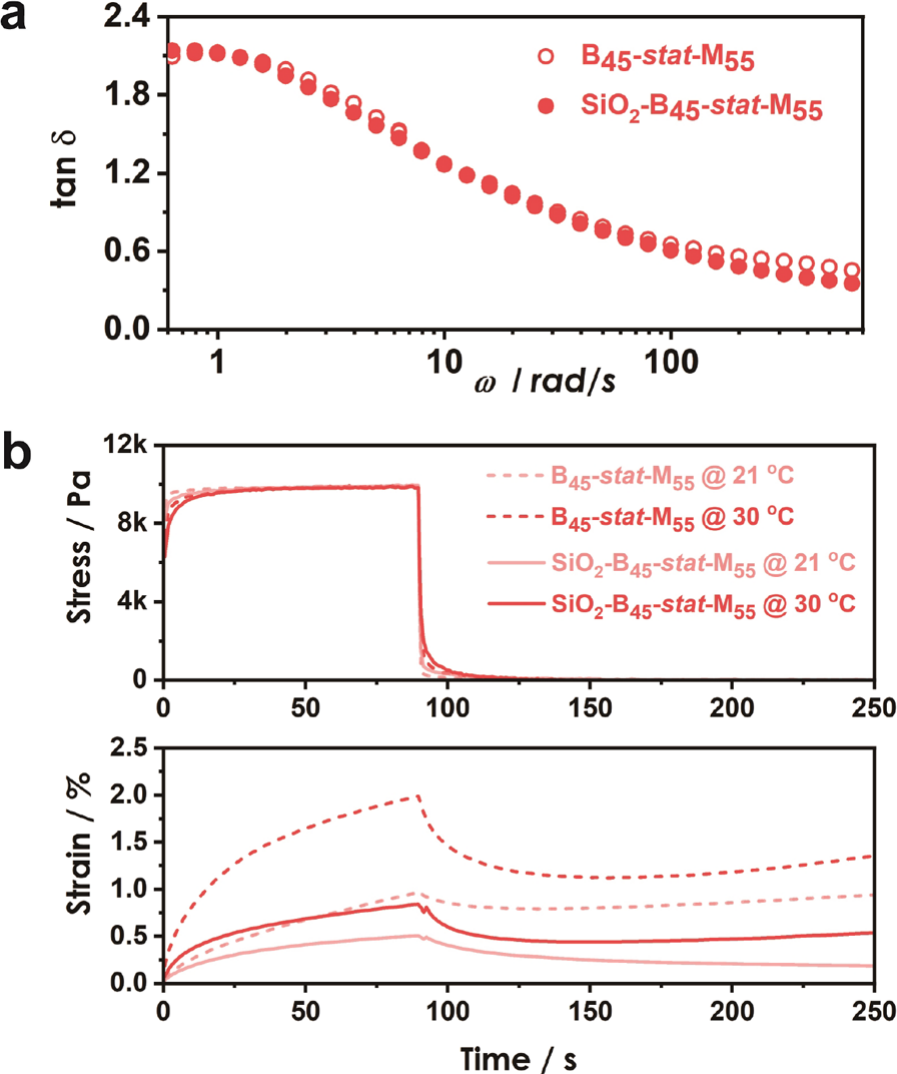
Local and macroscopic dynamical properties of statistical linear (B_45_-*stat*-M_55_) and brush (SiO_2_-B_45_-*stat*-M_55_) materials measured at 21 and 30 °C using dynamic mechanical analysis (a) and creep testing (b), respectively. (a) Near-identical loss tangent (tan δ) reveals similar local dynamics of linear and tethered chains (the relaxation frequency is estimated to be about 1 rad/s). (b) Step-function stress (10 kPa, upper plot) and resulting time-dependent strain (creep, lower plot) of linear (dashed lines) and brush particle (solid lines) hybrids. A significant reduction of strain along with the near complete recovery of the original shape upon removal of applied stress (at 21 °C) indicates an elastomer-type response in brush systems. Film dimensions for creep testing: width × length × height: 5 mm × 15 mm × 0.15 mm.

To evaluate the propensity of BA/MMA copolymer brush particle hybrids to exhibit shape-memory functionality, the local and macroscopic relaxation behavior was evaluated by dynamic mechanical analysis (DMA) and tensile creep testing, respectively. Figure 4a compares the loss tangent (tan δ) of statistical linear (B_45_-*stat*-M_55_) and brush particle (SiO_2_-B_45_-*stat*-M_55_) analogs, revealing near-identical relaxation dynamics. This is consistent with the similar *T*_g_’s of the linear (16 °C) and brush (19 °C) analogs and is interpreted to be a consequence of the similar segmental relaxation in both systems. The result thus confirms prior reports on homopolymer brush particle systems for which it was shown that the local dynamics of surface-tethered polymers is similar to the respective linear analog.^48,51−53^

To evaluate the macroscopic relaxation behavior of linear and brush systems, the time-dependent strain upon application of constant stress (10 kPa) of films was determined at 21 and 30 °C, respectively. A stress of 10 kPa was applied to ensure that both B_45_-*stat*-M_55_ and SiO_2_-B_45_-*stat*-M_55_ were in the linear deformation regime (as determined from tensile tests, Figure S2). Creep data as shown in Figure 4b revealed that the linear polymer analog (dashed lines) featured a significantly larger strain amplitude as well as nonrecoverable deformation, thus confirming a larger contribution of viscous flow. In contrast, the SiO_2_-B_45_-*stat*-M_55_ brush analog, despite featuring similar local dynamics, revealed (at 21 °C) a nearly complete recovery of the pristine shape, thus featuring a similar response to an elastomer-type polymer. The creep results provide direct experimental evidence in support of earlier claims that cooperative relaxations, unique to brush particle hybrids, result in the slowdown of diffusive displacements and flow across macroscopic lengthscales.^48,51,52,69−71^

**Figure 5:**
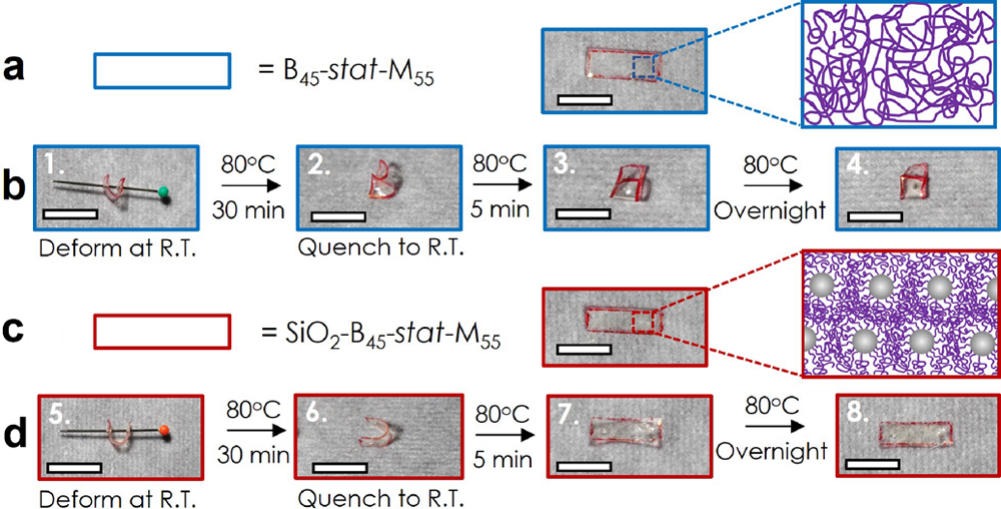
(a) Scheme for thermal stability of B_45_-*stat*-M_55_ at 80 °C for 30 min. Inset images: bulk film before and after thermal annealing. (b) U-bending shape-memory test for B_45_-*stat*-M_55_: 1. A B_45_-*stat*-M_55_ bulk film was bent to a U shape at 21 °C and fixed by a drawing pin. 2. The bent film was annealed at 80 °C for 30 min and quenched to 21 °C. 3. Reheating of the quenched film to 80 °C for 5 min. 4. Reheating of the quenched film to 80 °C overnight. (c) Scheme for thermal stability of SiO_2_-B_45_-*stat*-M_55_ at 80 °C for 30 min. Inset images: bulk film before and after thermal annealing. (d) U-bending shape-memory test for SiO_2_-B_45_-*stat*-M_55_: 5. A SiO_2_-B_45_-*stat*-M_55_ bulk film was deformed to a U shape at 21 °C and fixed by a drawing pin. 6. The bent film was annealed at 80 °C for 30 min and subsequently quenched to 21 °C. 7. Reheating of the quenched film to 80 °C for 5 min. 8. Reheating of the quenched film to 80 °C overnight. Bulk films’ edges are marked by red pen for better visibility. Scale bars equal 10 mm.

The slow relaxation in SiO_2_-B_45_-*stat*-M_55_ renders the brush particle hybrid an interesting candidate for facilitating shape-memory behavior. As elaborated before, shape-memory behavior is expected if the relevant relaxation modes are slow enough to be considered “static” and store enough strain energy to facilitate restoration of the permanent shape. While sufficient slowdown of relaxation is typically related to vitrification or the installment of a reversible network, we hypothesized that microstructure characteristics—such as extensive entanglement networks linked through quasi-static particle cores—could provide suitable constraints to macroscopic relaxation. Figure 5 compares the shape-memory characteristics of B_45_-*stat*-M_55_ and SiO_2_-B_45_-*stat*-M_55_ subjected to U-bend testing. U-bend shapes were prepared by annealing film samples bent to a curvature of 0.35 mm^–1^ for 30 min at 80 °C, followed by a quench to 21 °C at which both, linear and brush particle films, retained the bent shape. Shape-memory behavior was evaluated by reheating films to 80 °C. As depicted in Figure 5, the linear B_45_-*stat*-M_55_ did not show any indication of shape reversal after 12 h of annealing while the SiO_2_-B_45_-*stat*-M_55_ brush particle system folded back to the pristine (“flat”) film shape. The distinct behavior could be rationalized as a direct consequence of the different dynamical properties of linear and brush particle films, i.e., the more rapid stress relaxation in the case of the linear B_45_-*stat*-M_55_ resulted in the decay of stored strain energy and hence the stabilization of the U-bend shape. In contrast, the slow cooperative relaxation of brush particle films was able to retain the elastically stored strain energy over the tested time, thus providing a driving force for shape restoration at elevated temperatures.

**Figure 6:**
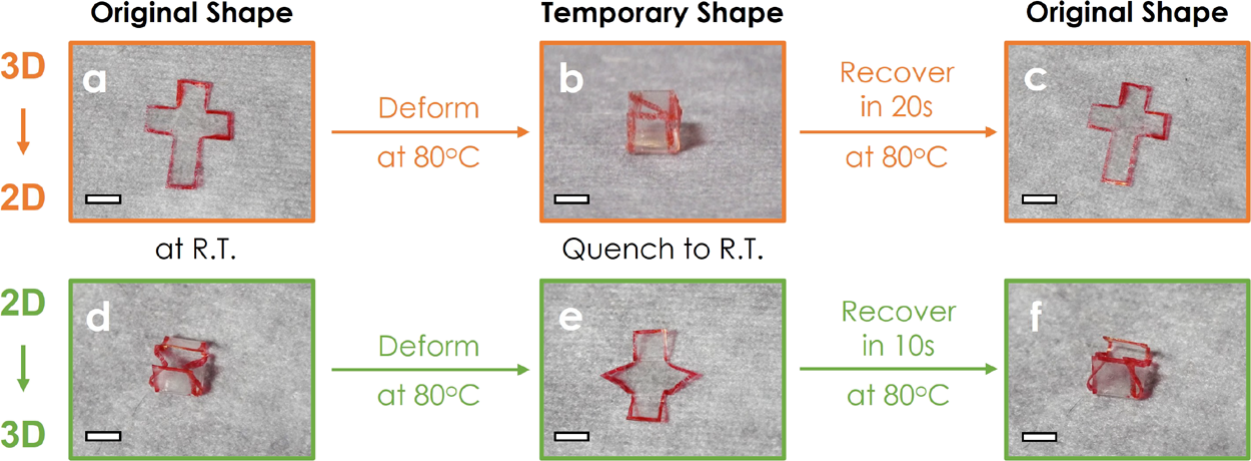
Shape-memory tests of SiO_2_-B_45_-*stat*-M_55_. (a) A bulk film was cut into specific shapes. (b) The cut film was folded into a hollow cube shape at 80 °C then quenched to R.T. (c) The cube was placed on an 80 °C heat plate, and it unfolded and recovered to its original shape. (d) A cut film was folded into a self-standing microgripper shape and then pressed under a heat press machine (80 °C, 100 psi) for 1 min. (e) The pressed film was unfolded into a flat shape at 80 °C and then quenched to R.T. (f) The flat shape film was placed on an 80 °C heat plate, and it automatically folded to its original shape.

The shape-memory behavior of SiO_2_-B_45_-*stat*-M_55_ was further evaluated using “kirigami”- and “origami”-inspired 3D structure transition such as the folding and unfolding of hollow cube structures (Figure 6). A manually constructed net shape of a hollow cube was cut from a film of SiO_2_-B_45_-*stat*-M_55_ with 0.15 mm thickness (the “kirigami”, Figure 6a). The reverse process is illustrated in Figure 6b,c. The net was folded into a 3D cube at 80 °C subsequently quenched to room temperature. The folded shape was maintained during all tested time periods. Unfolding was induced by heating the film to 80 °C, which caused the film to recover from 3D cube to the pristine 2D net shape after about 10 s (see Video S1). Additionally, the reverse shape recovery of the 2D net shape to a 3D microgripper shape was tested (Figure 6d–f, Video S2). This out-of-plane self-folding demonstrated that the shape-memory effect is not the consequence of viscosity or gravity but is due to the stored strain energy in deformed brush particle films. This confirmed that brush particles such as SiO_2_-B_45_-*stat*-M_55_ featured “recall-and-repair” capability (i.e., the concurrence of “shape-memory” and “self-heal” characteristics), which could find applications, for example, as 3D printing materials in areas like soft robotics that require a spatiotemporally localized response.^72−76^

## Conclusions

The assembly of BA/MMA-based copolymer brush particles provided a path toward hybrid materials with combined shape-memory and self-heal and shape-memory functionality (“recall-and-repair”). The effect of sequence and composition on self-healing in BA/MMA copolymer brush particle hybrids followed similar trends as for linear analogs. Most rapid restoration of mechanical properties was found for statistical sequence, which, as for linear analogs, was attributed to a more uniform microstructure. The increase of the high *T*_g_ (MMA) component provided a path to raise the material’s modulus while retaining self-heal ability. Creep testing (at 21 °C) revealed profound differences between linear and brush statistical copolymer materials. While linear copolymers featured substantial viscous flow when exposed to constant stress in the linear regime, permanent deformation (flow) was minimal for brush particle materials, which displayed a more rubbery-type response. Flow reduction was interpreted to be a consequence of slow cooperative relaxation due to the complex microstructure of brush particle hybrids in which long-range motions are constrained through entanglements and slow-diffusing particle cores. The rubbery-type response imparts BA/MMA copolymer brush material systems a dual “self-heal” and “shape-memory” capability, which could find application in the design of functional hybrid materials, e.g., for soft robotics, soft coating materials, and self-healable photonic materials. By leveraging the contribution of stored strain energy to promote structure restoration, this dual “recall-and-repair” functionality could also provide a path to enable shape-memory-assisted self-healing in higher modulus polymer materials, in damage scenarios that involve yield rather than complete bond scission. Evaluating “recall-and-repair” functionality, along with the elucidation of structure–property relationships in copolymer-based materials as well as the role of viscoelasticity, will be the subject of future work.

## Experimental Section

### Materials Synthesis

A series of linear copolymers and brush particles with various sequences and compositions were synthesized using activators regenerated by electron transfer (ARGET) atom transfer radical polymerization (ATRP) and SI-ATRP. Detailed feeding ratios and synthesis procedures are included in the Supporting Information.

### Materials Characterization

After the synthesis of materials, the grafted copolymers were first cleaved from the nanoparticle surface via hydrofluoric acid solution treatments and then the molecular weight and molecular compositions were tested by size exclusion chromatography (SEC) and nuclear magnetic resonance spectroscopy (NMR), respectively. Note that the silica nanoparticles were etched by hydrofluoric acid solution before measurements. The inorganic fraction of fully dried brush particles was measured by thermogravimetric analysis (TGA), and the grafting density was calculated via eq S1. Thermal properties like glass transition were performed by differential scanning calorimetry (DSC). To characterize mechanical properties, bulk films were first fabricated via casting linear copolymers and brush particle tetrahydrofuran (THF) solutions into Teflon molds, followed by vacuum annealing. The tensile test, creep behavior, damping property, and stress relaxations were carried out at the TA RSA-G2. Detailed characterization procedures are included in the Supporting Information.

## Supporting Information Available

Shape memory unfold (MP4)
Shape memory fold (MP4)
Details of materials, synthetic procedures, characterization procedures, derivative healing flow curves, strain–stress curves, and self-healing data (PDF)
