# Supplementary material for: Copolymer Brush Particle Hybrid Materials with “Recall-and-Repair” Capability
Source: Chem Mater. 2023 Aug 17;35(17):6990–7. doi: 10.1021/acs.chemmater.3c01234 (PMC10501442; doi:10.1021/acs.chemmater.3c01234)
Supplement: Supplementary file 3 — cm3c01234_si_003.pdf [file cm3c01234_si_003.pdf]

Supporting Information

Copolymer Brush Particle Hybrid Materials with 'Recall-and-Repair' Capability

Yuqi Zhao[†,§], *Hanshu Wu*[†,§], *Rongguan Yin*[‡], *Chenxi Yu*[†], *Krzysztof Matyjaszewski**[‡], *Michael R. Bockstaller**[†]

[†]Department of Materials Science & Engineering, Carnegie Mellon University, 5000 Forbes Avenue, Pittsburgh, Pennsylvania 15213, United States

[‡]Department of Chemistry, Carnegie Mellon University, 4400 Fifth Avenue, Pittsburgh, Pennsylvania 15213, United States

§ Y.Z. and H.W. contributed equally

Corresponding Author
* Krzysztof Matyjaszewski; Email: km3b@andrew.cmu.edu
* Michael R. Bockstaller; Email: bockstaller@cmu.edu

Supplementary Text

Materials

SiO_2-Br initiator was prepared as reported.[1] Ethyl α-bromoisobutyrate (eBiB, 98%, Sigma-Aldrich), anisole (99%, Aldrich), tetrahydrofuran (THF, 99%, VWR), methanol (99%, VWR), tris(2-dimethylaminoethyl)amine (Me_6TREN, 99%, Alfa), copper(II) bromide ($CuBr_2$, 99%, Aldrich), tin(II) 2-ethylhexanoate ($Sn(EH)_2$, 95%, Aldrich), N,N-dimethylformamide (DMF, certified, Fisher Chemical). Monomers: n-butyl acrylate (BA, 99%, Aldrich), methyl methacrylate (MMA, 99%, Aldrich) were purified by passing through a column filled with basic alumina to remove the inhibitor. Alumina (basic, Super I， 50-200μm, Sorbtech).

Synthesis of linear copolymer and particle brushes

Synthesis of linear PBA-statistical-PMMA copolymer and SiO_2-g-PBA-statistical-PMMA particle brushes.

Initiator (EBiB/SiO_2-Br), monomer: BA and MMA, solvents (anisole), $CuBr_2$, and Me_6TREN, molar ratios shown in supporting information, were mixed thoroughly in a sealed Schlenk flask, followed by degassing by bubbling with nitrogen. Then the $Sn(EH)_2$ was injected into the Schlenk flask to activate the catalyst complex, and the flask was immediately put into an oil bath set at the desired temperature. The conversion was monitored and controlled under 10% by ^{1}H-NMR. The final products were precipitated in cold methanol and then dissolved and stored in THF.

Molar ratios for synthesis of SiO_2-B_{52}-stat-M_{48}, SiO_2-B_{45}-stat-M_{55}, and B_{45}-stat-M_{55} via ARGET ATRP.

For SiO_2-B_{52}-*stat*-M_{48}, Initiator (SiO_2-Br, 0.2g), monomer: MMA (3.76 g, 4.0 mL) and BA (13.35 g, 15 mL), solvents (anisole 10 mL), $CuBr_2$ (0.005 g in 1 mL DMF), Me_6TREN (0.01 mL), and $Sn(EH)_2$ (0.06 g, 0.05mL).

For SiO_2-B_{45}-*stat*-M_{55}, Initiator (SiO_2-Br, 0.2g), monomer: MMA (4.70 g, 5.0 mL) and BA (12.46 g, 14 mL), solvents (anisole 10 mL), $CuBr_2$ (0.005 g in 1 mL DMF), Me_6TREN (0.01 mL), and $Sn(EH)_2$ (0.06 g, 0.05mL).

For B_{45}-*stat*-M_{55}, Initiator (eBiB, 0.004 mmol), monomer: MMA (3.29g, 3.5 mL) and BA (8.46 g, 9.5 mL), solvents (anisole 10 mL), $CuBr_2$ (0.005 g in 1 mL DMF), Me_6TREN (0.01 mL), and $Sn(EH)_2$ (0.06 g, 0.05mL).

Synthesis of SiO_2-g-PBA-grad-PMMA/SiO_2-g-PMMA-grad-PBA particle brushes.

Initiator (SiO_2-Br), monomer (BA/MMA), solvents (anisole), $CuBr_2$, and Me_6TREN, molar ratios shown in supporting information, were mixed thoroughly in a sealed Schlenk flask, followed by degassing by bubbling with nitrogen. Then the $Sn(EH)_2$ was injected into the Schlenk flask to activate the catalyst complex, and the flask was immediately put into an oil bath set at the desired temperature. Meanwhile, nitrogen pre-degassed MMA/BA monomer was injected to the reaction by syringe pump at the specific feeding rate. The conversion was monitored and controlled under 10% by ^{1}H-NMR. The final products were precipitated in cold methanol and then dissolved and stored in THF.

Molar ratios for synthesis of SiO_2-B_{51}-grad-M_{49} and SiO_2-M_{49}-grad-B_{51} via ARGET ATRP.

For SiO_2-B_{51}-*grad*-M_{49}, Initiator (SiO_2-Br, 0.2g), monomer: MMA (6.2 g, 6.6 mL) and BA (12.46 g, 14 mL), solvents (anisole 10 mL), $CuBr_2$ (0.005 g in 1 mL DMF), Me_6TREN (0.01 mL), and $Sn(EH)_2$ (0.06 g, 0.05mL). MMA feeding rate: 2.2 mL/h.

SiO_2-M_{49}-*grad*-B_{51}, Initiator (SiO_2-Br, 0.2g), monomer: MMA (2.35 g, 2.5 mL) and BA (18.69 g, 21 mL), solvents (anisole 10 mL), $CuBr_2$ (0.005 g in 1 mL DMF), Me_6TREN (0.01 mL), and $Sn(EH)_2$ (0.06 g, 0.05mL). BA feeding rate: 7 mL/h.

Nuclear Magnetic Resonance Spectroscopy (NMR)

Conversion of polymerization was monitored by ^{1}H NMR on a Bruker Advance 500 MHz NMR instrument in $CDCl_3$ at room temperature.

Fabrication of a bulk film

THF dissolved linear copolymers and particle brushes dispersion solutions were transferred into 15 mm × 5 mm rectangular Teflon molds. The solvent was slowly evaporated over 48 h at room temperature generating transparent nanocomposite films with a thickness of 0.1-0.2 mm. The residual solvent was removed from the bulk films by transferring them to a vacuum oven at 120 °C for 24 h.

Size Exclusion Chromatography (SEC)

Number-average molecular weights (Mn) and molecular weight distributions (MWD) of samples were determined by size exclusion chromatography (SEC). The SEC was conducted with an Agilent 1260 Iso pump and Waters 410 differential refractometer using PSS columns (Styragel 105, 103, 102 Å) with THF as an eluent at 35 ºC and at a flow rate of 1 mL min^{-1}. Linear PMMA standards were used for calibration. Toluene was used as internal standards for the system.

Differential Scanning Calorimetry (DSC)

The glass transition temperature (T_g) of materials were measured by differential scanning calorimetry (DSC) with TA Instrument QA-2000. The same procedure involving the following steps: (1) Equilibrate at 25.00 °C, (2) Isothermal for 1.00 min, (3) Ramp 20.00 °C/min to -90.00

°C, (4) Isothermal for 1.00 min, (5) Ramp 20.00 °C/min to 160.00 °C, (6) Isothermal for 1.00 min, (7) repeat steps 3~6 twice, (8) Jump to 25.00 °C. The DSC data were analyzed with a TA Universal Analysis instrument, and T_g was directly acquired.

Thermogravimetric Analysis (TGA).

TGA with TA Instruments 2950 was used to measure the fraction of SiO_2 in the hybrids. The data were analyzed with TA Universal Analysis. The heating procedure involved four steps: (1) jump to 120 ºC; (2) hold at 120 ºC for 10 min; (3) ramp up at a rate of 20 ºC/min to 800 ºC; (4) hold for 5 min.

Grafting density was calculated using formula (S1).

$$\sigma_{\mathrm{TGA}} = \frac{(1-f_{\mathrm{SiO2}})N_{\mathrm{Av}}\,\rho_{\mathrm{SiO2}}\,d}{6\,f_{\mathrm{SiO2}}\,M_n} \qquad \text{(S1)}$$

where f_{SiO2} is the SiO_2 fraction measured by TGA, N_{Av} is the Avogadro number, ρ_{SiO2} is the density of SiO_2 nanoparticles (2.2 g/cm3), d is the average diameter of SiO_2 nanoparticles (15.8 nm), Mn is the overall number-average MW of the cleaved polymer brushes.

Mechanical Properties Analysis

Tensile test: the linear copolymer bulk films are tested in the tensile mode by using TA RSA-G2. The film thickness was between 150-200 µm. The samples were stretched at a constant tensile rate of 0.05 s^{-1} at room temperature.

Dynamic mechanical analysis (DMA): Damping property measurement: The damping property was measured through dynamic mechanical analysis (DMA, TA RSA-G2) in a frequency range of 0.1-100 Hz at room temperature, with application of 0.1% strain. All the samples were tested at least three times for consistency.

Creep test: Creep experiments were performed on pristine specimens with applied stress of 10 kPa for 90 seconds at room temperature (TA RSA-G2), followed by a recovery time of 180 seconds in which stress was removed.

Self-healing Test

A bulk film was severed, then physically reattached within 1 min at 50 ºC, and allowed to self-healing for a specific time under 100 ºC. After that, the same film after healing was characterized by a tensile test as described above. Then, compared the pristine samples and calculated the recovery ratios.

Shape Memory Test

For the U-bending test[26], a 15 mm × 5 mm rectangular bulk film with flat permanent shape was bent to U-shape and fixed by a drawing pin at 80 ºC for 30 minutes subsequently quenching to room temperature. Then the fixed U-shape film was reheated at 80 ºC for specific time for shape recovery. For 3D shape memory test, the details were described in results and discussion section.

Transmission Electron Microscopy (TEM).

TEM was carried out using a Thermo Fisher Themis 200 G3 electron microscope. The diameters and inter-particle distances of the SiO_2 nanoparticles were determined from statistical analysis of the TEM micrographs using MatLab software.

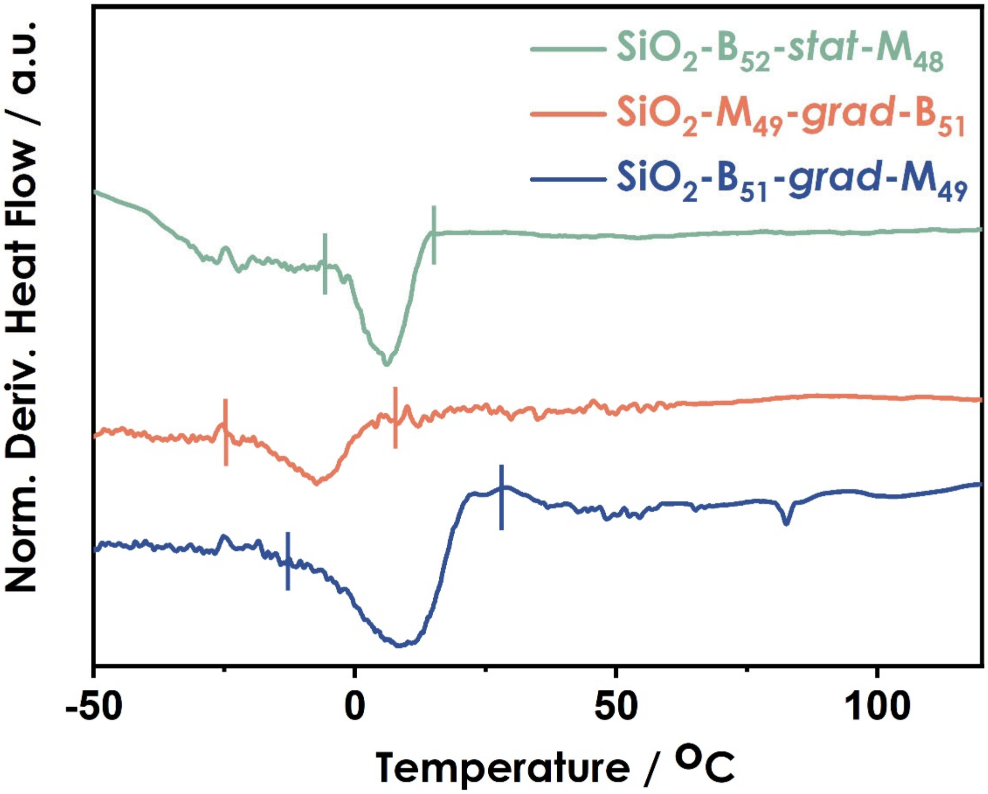

Figure S1. Normalized derivative DSC heat flow curves. The onset and offset points of the glass transitions are highlighted in the figures. Measurements were performed at the third cooling cycle at a heating rate of 20 ºC/min

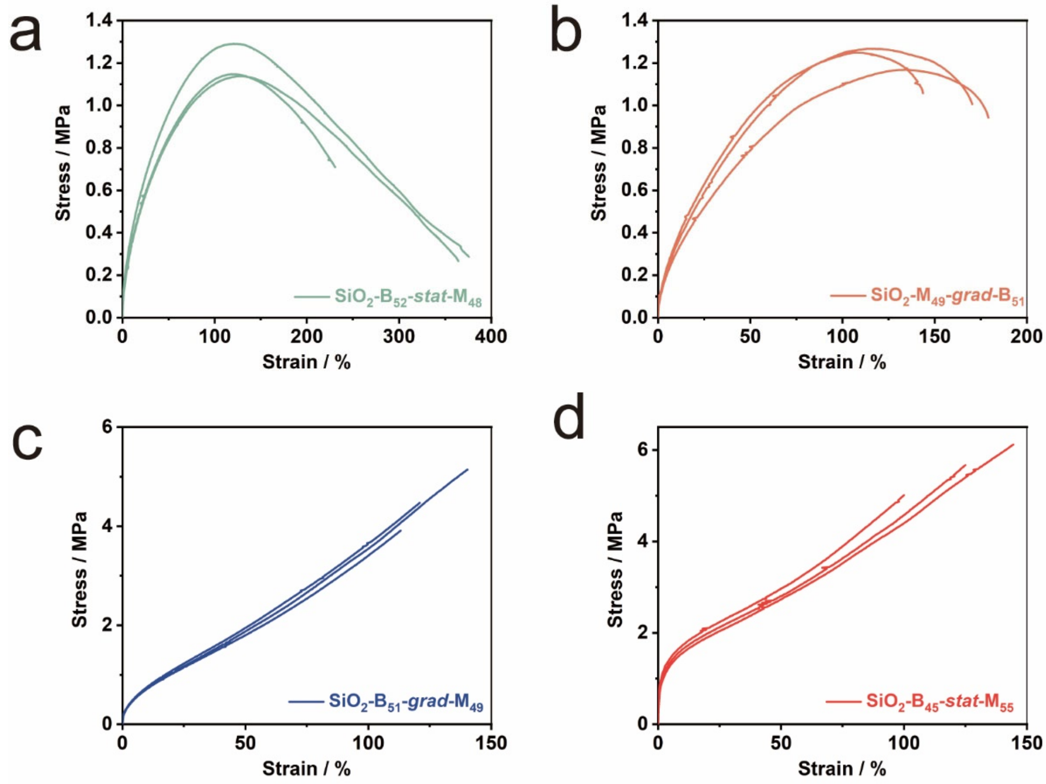

Figure S2. Strain-stress curves: (a) SiO_2-B_{52}-*stat*-M_{48}, (b) SiO_2-M_{49}-*grad*-B_{51}, (c) SiO_2-B_{51}-*grad*-M_{49}, (d) SiO_2-B_{45}-*stat*-M_{55}. All samples were measured three times with different bulk films as shown in same color lines in the figures.

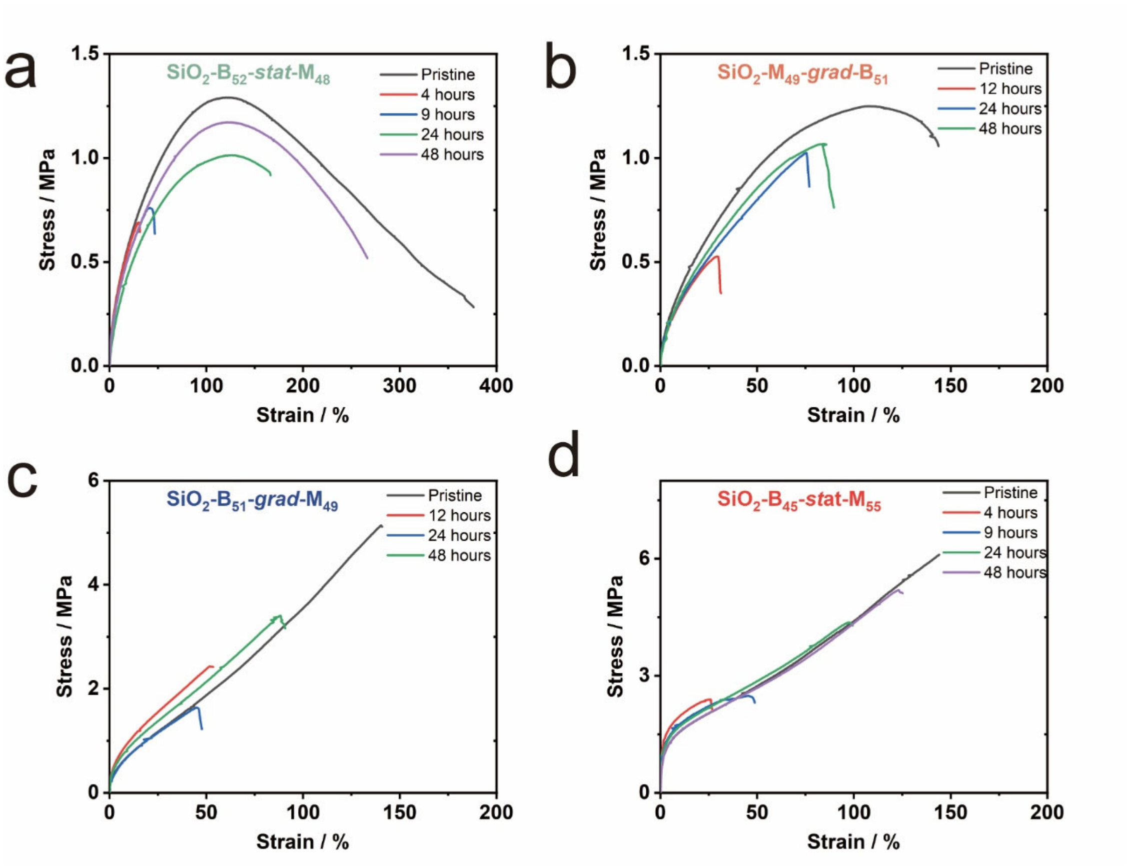

Figure S3. Strain-stress curves for pristine and damaged-and-healed films: (a) SiO_2-B_{52}-*stat*-M_{48}, (b) SiO_2-M_{49}-*grad*-B_{51}, (c) SiO_2-B_{51}-*grad*-M_{49}, (d) SiO_2-B_{45}-*stat*-M_{55}.

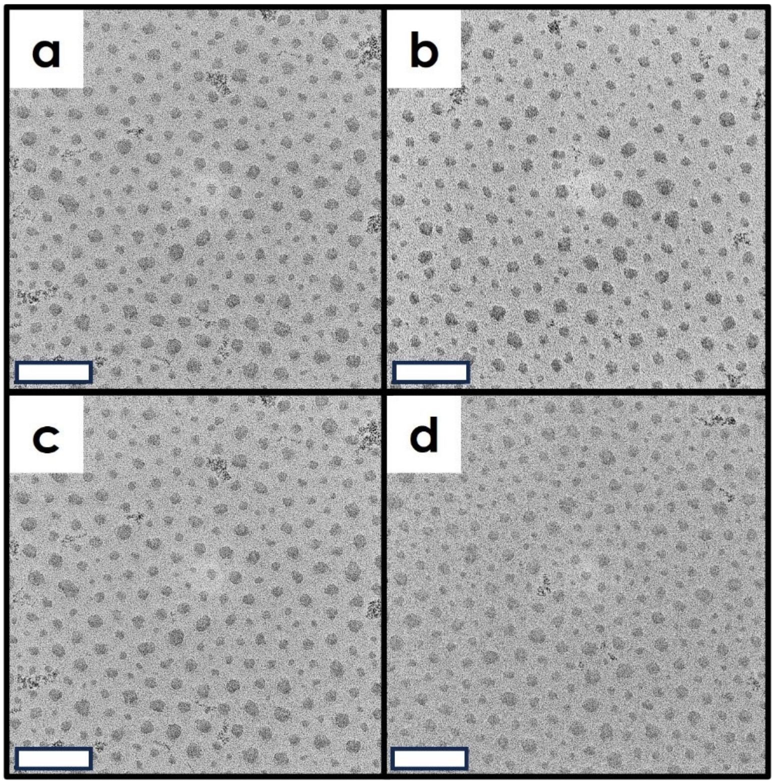

Figure S4. TEM images of SiO_2-g-P(BA-stat/grad-MMA) (a) SiO_2-B_{52}-*stat*-M_{48}, (b) SiO_2-B_{51}-*grad*-M_{49}, (c) SiO_2-M_{49}-*grad*-B_{51}, (d) SiO_2-B_{45}-*stat*-M_{55}, scale bar is 100 nm. Particle diameter determined from analysis of electron micrographs $d \sim 15.54 \pm 3.7$ nm.

Video S1

Shape memory unfold. Shape memory process for a SiO_2-B_{45}-*stat*-M_{55} bulk film. The sample unfolded automatically from hollow cube 3D shape to corresponding net 2D shape. Heat plate temperature is 80 ºC.

Video S2

Shape memory fold. Shape memory process for a SiO_2-B_{45}-*stat*-M_{55} bulk film. The sample folded automatically from a net 2D shape to a corresponding self-standing microgripper shape. Heat plate temperature is 80 ºC.

Reference

[1] Jiajun Yan, Xiangcheng Pan, Michael Schmitt, Zongyu Wang, Michael R. Bockstaller, and Krzysztof Matyjaszewski. *ACS Macro Letters* **2016** 5 (6), 661-665
